# Therapeutic efficacy of Chinese patent medicine containing pyrite for fractures: A protocol for systematic review and meta-analysis

**DOI:** 10.1097/MD.0000000000032267

**Published:** 2022-12-09

**Authors:** Su Hyeon Choi, Eun-Young Nam, Ji Hye Hwang

**Affiliations:** a College of Korean Medicine, Gachon University, Seongnam, Republic of Korea; b Mimi Korean Medicine Clinic, Seoul, Republic of Korea; c Department of Acupuncture and Moxibustion Medicine, College of Korean Medicine, Gachon University, Seongnam, Republic of Korea.

**Keywords:** Chinese patent medicine, fracture, protocol, pyrite, pyritum, systematic review

## Abstract

Fractures are a condition in which bone continuity is lost or linear deformity occurs. They are a worldwide public health problem and a significant economic burden. The purpose of this study is to analyze the efficacy of Chinese patent medicine containing pyrite (CPMP) through systematic review and meta-analysis of fracture clinical data.

**Methods:**

A literature search will be carried out from the inception of CPMP studies to September 2022 using EMBASE, PubMed, Cochrane Central Register of Controlled Trials, China National Knowledge Infrastructure, Korean Studies Information Service System, National Digital Science Library, and Oriental Medicine Advanced Searching Integrated System. Randomized controlled trials which include CPMP will be considered as eligible regardless of the type of fracture. After screening the literature, extracting the data, and assessing the risk of bias from the included studies, a meta-analysis will be performed using Review Manager version 5.4.

**Results:**

This study is expected to provide evidence for the efficacy and safety of CPMP for fractures.

**Conclusion:**

Our findings will provide evidence to determine whether CPMP can be an effective intervention for patients with fractures, which would expand the possible treatment options.

## 1. Introduction

A fracture is a bone that is broken or cracked, and the term refers medically to a condition in which bone continuity is lost or linear deformation occurs. In most cases, fractures are accompanied by not only bone damage, but also damage to soft tissues such as muscles and ligaments around the fracture site, nerves, blood vessels, and skin.^[1]^ In 2019, there were 178 million new fractures (an increase of 33.4% since 1990) and 455 million cases of acute or long-term fractures (increased by 70.1% since 1990) around the world. To put the disability burden of fractures in the context of other diseases and injuries, fractures were between the second-leading and fourth-leading cause of years of healthy life lost due to disability (YLDs) for the 10 countries and territories with the highest age-standardized rates of fractures in 2019.^[2]^ Bone fractures are a public health issue around the world and pose a serious economic burden,^[3,4]^ especially for people with osteoporosis.^[5]^ Fractures can lead to work absence, decreased productivity, disability, impaired quality of life, health loss, and high health-care costs and they are a major burden to individuals, families, societies, and health-care systems.^[6–8]^

The conventional methods for fracture treatment are reduction, external fixation, and internal fixation, but they depend on the site and the conditions.^[9]^ Some factors such as nutritional deficiencies, smoking, alcoholism, diabetes, some prescribed medicine like NSAIDs, low bone density, and osteoporosis may cause complications including delayed union or infections after treatment.^[10–12]^ Also, long-term treatment for a fracture limits the movement of a patient, which may lead to pneumonia or deep vein thrombosis, lowered body function maintenance and reduced quality of life.^[13,14]^ Therefore, in order to minimize the side effects and make it faster to recover the body’s functions, post-fracture treatment and care is important.

Mineral drugs are an important constituent of traditional Chinese medicine (TCM).^[15]^ Pyrite (*Pyritum*) has been used as a mineral traditional medicine in Korea and China for thousands of years. It has mainly been prescribed to promote bone formation, heal fractures, eliminate blood stasis, and relieve pain. To ensure clinical safety and efficacy, it is usually prescribed in a calcined or processed form.^[1,16–18]^ Regarding the use of pyrite for healing fractures, studies have reported on the biochemical, histomorphological, morphometric, and immunohistological methods for TGF-β1 related to fracture healing,^[18]^ and on the sequential changes in inflammatory response and osteoblast activity at the fracture site.^[17]^ In traditional Korean medicine (TKM), pyrite is often used alone or as part of a combination treatment in clinical practice, but there have been few clinical studies.^[19]^

On the other hand, several clinical studies using pyrite have been reported in TCM, and it is particularly used in the form of Chinese patent medicine containing pyrite (CPMP).^[20]^ In recent years, many effective herbal medicines and compound prescriptions have been made into Chinese patent medicines (CPMs), which are widely used in clinical practice and have significant effects.^[21]^ CPM uses TCM for raw materials and formulates them with the theory of TCM.^[22]^ In China, CPMs of the same name have the same proportions of ingredients, and are manufactured according to the monograph of the Pharmacopoeia of the People’s Republic of China (PPRC) for that particular formula, which is mandated by Chinese law.^[23]^ CPM has the same efficacy in treating a given disease as a TCM decoction, but it saves the time of decoction and it is easy to use clinically and safely due to laws and regulations.^[22]^ There were 10 types of CPMP listed in the Chinese Pharmacopoeia in 2020 and 26 types in the Newly Edited National Chinese Traditional Patent Medicines with 3 overlapping cases. Although several CPMPs have been reported to be used for fracture treatment,^[20]^ a comparative analysis through a systematic literature review on the efficacy of CPMPs in the treatment of fractures has not been performed. Therefore, this study intends to systematically review and analyze the currently available literature to secure evidence on the efficacy and safety of CPMP for fractures.

## 2. Methods

### 2.1. Study registration

The protocol for this systematic review was registered on the *International Prospective Register of Systematic Reviews* (*PROSPERO*) (Registration number: CRD42022366793). The current protocol for this review complies with the Preferred Reporting Items for Systematic Reviews and Meta-Analyses (PRISMA) protocols.^[24]^

### 2.2. Inclusion criteria

#### 2.2..1. Types of studies.

Prospective randomized controlled trials (RCTs) evaluating the therapeutic effect of CPMP for various fractures will be included in this review. Both treatment with CPMP alone and concurrent treatment with other therapies will be considered acceptable if only CPMP is applied to the intervention group and any other treatment is provided equally to both the intervention and control groups. Non-randomized trials, animal or cell studies, literature research, and case studies will be excluded. No language restrictions will be imposed.

#### 2.2..2. Types of participants.

The study will include all participants with a definitively diagnosed fracture, regardless of the type of fracture or the fracture site. There will be no restrictions on age, sex, race or nationality.

#### 2.2..3. Types of interventions and controls.

For patients with fractures, RCTs that include CPMP as the sole treatment or as an adjunct to other treatments will be included, as long as the RCTs including other treatments provide the same treatment to the control and intervention groups. Parenteral dosing studies such as external use of CPMP will be excluded. Trials with any type of control intervention compared to CPMP will be included.

#### 2.2..4. Types of outcome measures.

##### 2.2..4..1. Primary outcomes.

Efficacy rates will be measured as primary outcomes. These will include treatment effective rate, callus growth rate, bone union time, and edema disappearance time.

##### 2.2..4..2. Secondary outcomes.

The secondary outcomes will be assessed pain reduction by the visual analog scale and pain disappearance time. Erythrocyte sedimentation rate, hematocrit (HCT), and plasma viscosity will also be evaluated by blood test results.

### 2.3. Search strategy

Two independent authors will conduct a literature search within databases from the inception of CPMP studies to September 2022. The databases searched will be EMBASE, PubMed, Cochrane Central Register of Controlled Trials, China National Knowledge Infrastructure, Korean Studies Information Service System, National Digital Science Library, and Oriental Medicine Advanced Searching Integrated System. RCTs which includes CPMP will be considered as eligible regardless of the type of fracture. No language restrictions will be imposed.

The search will be conducted by using the keywords “pyrite,” “pyritum,” and “zirantong.” CPMP searches will be conducted based on CPMs included in the Chinese Pharmaceutical Dictionary and Newly Edited National Chinese Traditional Patent Medicines.

### 2.4. Data collection and analysis

#### 2.4..1. Data extraction and management.

**Figure 1.**
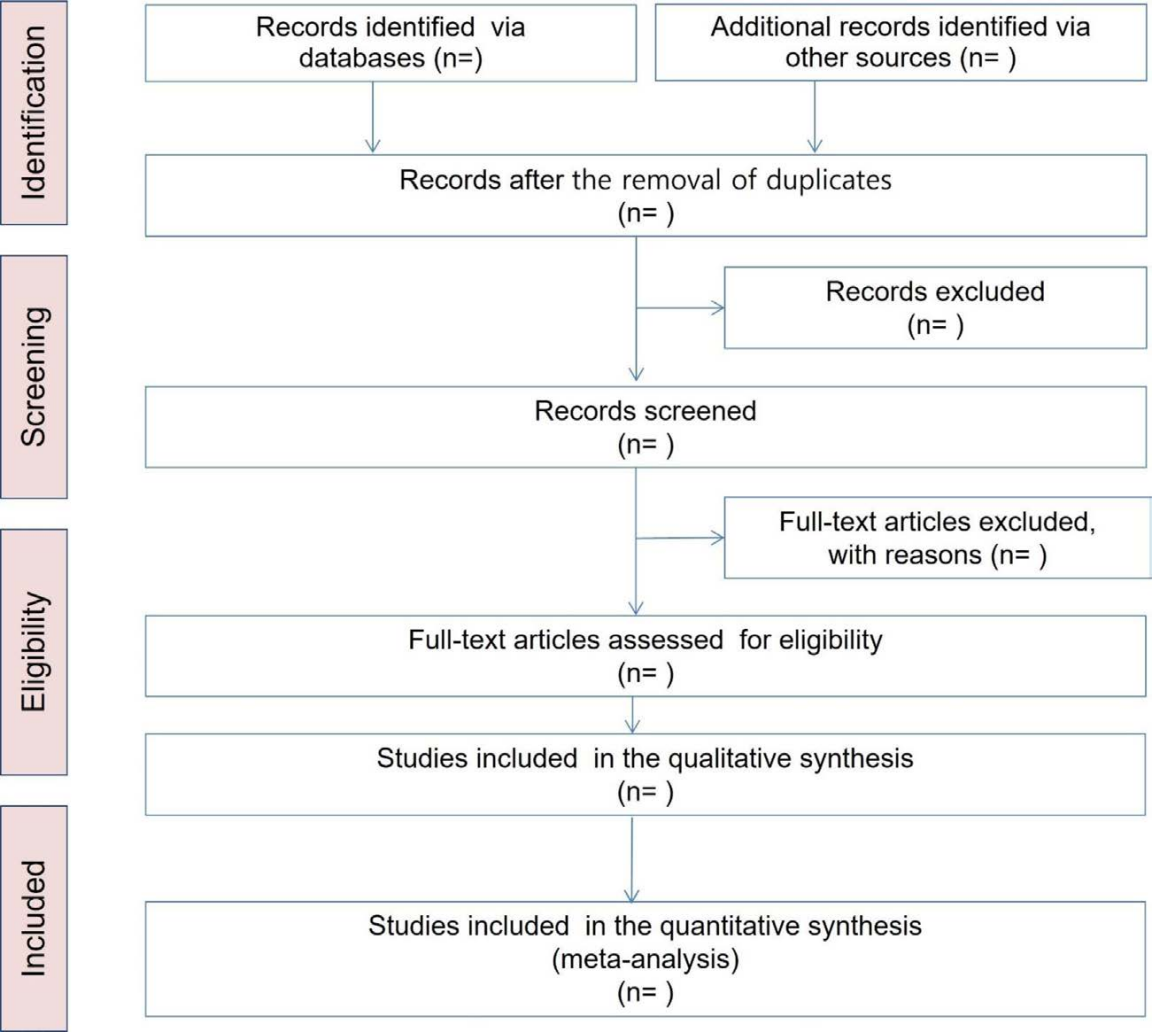
PRISMA flow diagram.

All searched articles will be reviewed to evaluate their eligibility for inclusion. In cases of uncertainty, the authors will be contacted for further information. All identified articles will be managed in Endnote (Clarivate Analytics, New York, NY) to evaluate the eligibility for inclusion. Articles will be screened and eliminated based on the inclusion and exclusion criteria, and duplicate articles will be manually removed. After the selection of studies, 2 researchers will conduct the data extraction independently with a data extraction form. The extracted data will include the author, year of publication, study design, participants (age and gender), sample size, diagnostic criteria of fracture, classification of diseases, detailed information of intervention and control treatment (treatment method, treatment duration, dosage, etc.), main outcome measurements, results, adverse effects, and blinding method. Any disagreements or uncertainties during the review will be resolved by discussion and consensus among all researchers (Fig. 1).

#### 2.4..2. Assessment of risk of bias in included studies.

Risk of bias will be assessed using the Cochrane Handbook risk of bias assessment tool version 5.1.0 to take into account random sequence generation, allocation concealment, blinding of participants and personnel, blinding of outcome assessment, incomplete outcome data, selective reporting, and other sources of bias. The results of the assessments will be presented using “L” to indicate a low risk of bias, “U” to indicate an uncertain risk of bias, and “H” to indicate a high risk of bias. The quality assessment will be evaluated independently by 2 people. If there is an evaluation disagreement between the 2 people, the literature will be checked again. If there is a continuous disagreement, an agreement will be reached through discussion with a third party. If information from an article cannot be obtained to do the assessment, the corresponding author will be contacted for that information.

### 2.5. Data synthesis and analysis

Differences between the intervention and control groups will be assessed. Mean differences (MDs) with 95% confidence intervals (CIs) will be used to measure the effects of treatment for continuous data. Other forms of data will be converted into MDs. For outcome variables on different scales, standard MDs with 95% CIs will be used. For dichotomous data, treatment effects as relative risks with 95% CIs will be presented. Other binary data will be converted into relative risk values.

All statistical analyses will be conducted using Cochrane Collaboration’s Review Manager version 5.4 (The Nordic Cochrane Centre, The Cochrane Collaboration, Copenhagen, Denmark) for Windows. The corresponding authors of studies with missing information will be contacted whenever possible to acquire and verify the data. When appropriate, data across studies will be pooled to conduct a meta-analysis using fixed or random effects. GRADEpro from Cochrane Systematic Reviews will be used to create a summary of findings table.

If the necessary data are available, subgroup analysis will be carried out for the different types of therapies available and compared with CPMP.

The statical heterogeneity will be assessed by *I*^2^ and P values based on 95% CIs, and *I*^2^ < 25% will be considered as low heterogeneity, 25% to 50% as moderate, >50% as high heterogeneity, and *P* < .05 will be considered as significant. If the heterogeneity is significant, meta-analysis will use random effects, but insignificant heterogeneity will use fixed effects. The subgroup analysis will be evaluated using the X2 test, and a sensitivity analysis will be conducted to determine the robustness of merged results by deleting low-quality studies. The publication bias will be evaluated by funnel plots.

### 2.6. Ethics and dissemination

As the study will review published literature, there is no patient recruitment and no personal data collection, thus, ethical approval is not required. The results of this systematic review will be published in a peer-reviewed journal and disseminated electronically and in print. In order to inform and guide healthcare practices, the review will be updated.

## 3. Discussion

Bone is a complex material composed of organic and inorganic components such as hydroxyapatite, collagen, proteoglycans, noncollagenous proteins, and water. Bone fractures are common in individuals with osteoporosis or osteogenesis imperfecta, or individuals who routinely perform heavy physical activities.^[25,26]^ Unlike soft tissues, bone fracture healing is a complicated process involving various factors at the cellular and molecular levels.^[26,27]^ Despite technological advancements made by understanding the factors responsible for fracture healing, 5 to 20% of patients still suffer from delayed healing or nonunion in long bone fractures.^[26,28]^

Alternative forms of treatment have developed worldwide over the past few decades. Herbal medicines are currently in demand globally and their popularity is growing. In developing countries, it was estimated that 80% of the population is still dependent on traditional herbal medicines.^[29]^ Furthermore, herbal medicines have been widely used in clinical practice to treat bone diseases for thousands of years. According to TCM and TKM theory, the pathological syndromes of a bone fracture include redness and pain in the fracture area, swelling and stagnation of circulation, and slow bone healing. TCM and TKM suggest that fracture healing can be improved with herbs that control inflammation, promote blood circulation, and stimulate bone regeneration. These herbs are known to have fewer side effects, be more cost-effective, and more suitable for long-term use compared to chemically synthesized drugs.^[30]^ However, it is difficult to find mentions of mineral traditional medicines including pyrite in some of the studies on traditional herbal medicines or remedies for fracture treatment.^[29–31]^

This study will systematically evaluate the fracture treatment efficacy and safety of CPMP in order to provide a reference for clinical traditional medicine selection for fractures. There are clinical studies utilizing CPMP for bone fracture treatment in recent years that have found significant therapeutic effects, but there are no relevant systematic reviews or meta-analysis reports. Therefore, our study will evaluate the current evidence for the effectiveness of CPMP of TCM to provide more treatment options for patients with fractures, and encourage more experts and doctors to carry out more research in the future. This evidence will be useful to patients, practitioners, and health policy makers. Patients with fractures will be able to receive appropriate pyrite containing herbal medicine treatments, and practitioners will be able to provide better informed decisions for treatment. In addition, this study will contribute to the accumulation of evidence for the use of pyrite only and pyrite combination prescriptions in complementary therapies for better management of bone-related diseases and will serve as a basis for the design of clinical studies.

## Author contributions

**Conceptualization:** Ji Hye Hwang, Su Hyeon Choi.

**Formal analysis:** Su Hyeon Choi, Ji Hye Hwang.

**Funding acquisition:** Ji Hye Hwang.

**Investigation:** Su Hyeon Choi, Ji Hye Hwang.

**Methodology:** Eun-Young Nam, Su Hyeon Choi.

**Writing – original draft:** Ji Hye Hwang, Su Hyeon Choi.

**Writing – review & editing:** Ji Hye Hwang, Eun-Young Nam.
